# Deep learning to predict elevated pulmonary artery pressure in patients with suspected pulmonary hypertension using standard chest X ray

**DOI:** 10.1038/s41598-020-76359-w

**Published:** 2020-11-17

**Authors:** Kenya Kusunose, Yukina Hirata, Takumasa Tsuji, Jun’ichi Kotoku, Masataka Sata

**Affiliations:** 1 Department of Cardiovascular Medicine, Tokushima University Hospital, 2-50-1 Kuramoto, Tokushima, Japan; 2 Ultrasound Examination Center, Tokushima University Hospital, Tokushima, Japan; 3 Department of Radiological Technology, Graduate School of Medical Care and Technology, Teikyo University, Tokyo, Japan

**Keywords:** Cardiology, Diagnosis, Medical imaging

## Abstract

Accurate diagnosis of pulmonary hypertension (PH) is crucial to ensure that patients receive timely treatment. We hypothesized that application of artificial intelligence (AI) to the chest X-ray (CXR) could identify elevated pulmonary artery pressure (PAP) and stratify the risk of heart failure hospitalization with PH. We retrospectively enrolled a total of 900 consecutive patients with suspected PH. We trained a convolutional neural network to identify patients with elevated PAP (> 20 mmHg) as the actual value of PAP. The endpoints in this study were admission or occurrence of heart failure with elevated PAP. In an independent evaluation set for detection of elevated PAP, the area under curve (AUC) by the AI algorithm was significantly higher than the AUC by measurements of CXR images and human observers (0.71 vs. 0.60 and vs. 0.63, all p < 0.05). In patients with AI predicted PH had 2-times the risk of heart failure with PH compared with those without AI predicted PH. This preliminary work suggests that applying AI to the CXR in high risk groups has limited performance when used alone in identifying elevated PAP. We believe that this report can serve as an impetus for a future large study.

## Introduction

Accurate diagnosis of pulmonary hypertension (PH) is crucial to ensure that patients receive timely treatment for a progressive clinical course^1^. Although approaches for accurate diagnosis of PH may avoid development to symptomatic heart failure, a low-cost and noninvasive screening tool does not exist in the clinical setting. As a result, several groups have sought to identify minimally invasive or noninvasive approaches to identifying patients with PH. The American College of Chest Physicians has recommended obtaining a chest X-ray (CXR) in patients who are suspected of having PH, to reveal features supportive of a diagnosis of PH^2^. However, it is well known that the sensitivity and specificity are low. Currently, the recommended test for screening is echocardiography; however, the test requires intensive training and highly qualified technical staff and is relatively expensive^3^. Although the multi-modality assessment plays a central role for the assessment of PH^4^, the accessibility of diagnostic modalities is sometimes limited in the many remote areas and facilities. With this in mind, we may need a widely usable strategy for identifying a high-risk cohort requiring appropriate right heart catheter (RHC) procedures.

Artificial intelligence (AI), using neural networks, has been applied to sophisticated recognition of subtle patterns in digital data in medical fields^5,6^. A family of algorithms has led to state-of-the art improvement in word recognition, visual object recognition, object detection, etc^7,8^. Recently, simple digital data (e.g., electrocardiogram) can identify asymptomatic left ventricular dysfunction at baseline and during follow-up using AI^9^. Thus, we hypothesized that application of AI to the CXR could identify a high-risk cohort of PH and stratify the risk of heart failure hospitalization with PH. To test this hypothesis, we created, trained, validated, and then tested a neural network.

## Materials and methods

### Study population

Flow chart of patient recruitment was shown in Supplemental Fig. S1. We retrospectively enrolled a total of 900 patients with paired RHC and chest CXR referred to our laboratory for evaluation of pulmonary hemodynamics. These consecutive patients had experienced symptoms or signs of heart failure with suspected PH between October 2009 and December 2018. In our hospital, if the findings for PH were observed in CXR (1: frontal chest radiograph shows a prominent main pulmonary artery, 2: dilated right interlobar artery, and 3: pruning of peripheral pulmonary vascularity) and echocardiography (1: elevated tricuspid regurgitation velocity, 2: dilatation of the right ventricle, 3: dilatation of inferior vena cava), we recommended the right heart catheter to assess the hemodynamic parameters. The first RHC-CXR data pair from patients with RHC and CXR performed within a one-day interval constituted the analysis data set. We excluded patients with emergency cardiac catheterization and unstable clinical condition due to the data quality. To test the AI model compared with measurements of CXR images and human observers, we have gathered a separate group of 90 consecutive patients with RHC-CXR data between January 2019 and June 2019. To overcome the issue of generalizability, we have gathered an independent external validation group of 55 patients with suspected PH by CXR or echocardiography who were referred for right heart catheterization between January 2020 and July 2020. The Institutional Review Board of the Tokushima University Hospital approved the study protocol (no. 3217-3). All patients signed an informed consent. All methods were in accordance with the relevant guidelines and regulations.

### Right heart catheterization

We used the data from RHC because RHC is the gold standard for the diagnosis and hemodynamic assessment of pulmonary hypertension. RHC was performed using a Swan-Ganz catheter. Pressure measurements were obtained at end-expiration while the patients were supine. The following hemodynamic parameters were recorded: mean pulmonary artery wedge pressure (PAWP), mean pulmonary artery pressure (PAP), mean right atrial pressure, and cardiac output (CO). CO was measured using the indirect Fick equation. Pulmonary vascular resistance (PVR) was defined as (mean PAP − mean PAWP)/CO. The diagnosis of PH was performed using hemodynamic measurements according to the most recent World Symposium standards: mean PAP > 20 mmHg^10,11^.

### Eyeball assessment

Visual assessments were interpreted by consensus of 10 physicians who were blinded to RHC data using CXR images based on the radiographic findings of PH: (1) frontal chest radiograph shows a prominent main pulmonary artery, (2) dilated right interlobar artery, and (3) pruning of peripheral pulmonary vascularity in the guideline and report^12,13^.

### Measurements of chest X-rays

CXR images were measured by a physician who were blinded to RHC data. In the CXR of all subjects, four measurements were done (Supplemental Fig. S2): (1) Measurement of the widening of hilum from the most lateral visible border of hilum to the other lateral border (Hilum). (2) Projection of the right heart border (PRHB) that consists of the distance from the right visible border of the right side of the heart up to the midline of the thorax. (3) Ratio of hilar widening to the chest widening that consists of widening of the interior chest cage in the level of hilum (Hilum/chest ratio). (4) Addition of PRHB to hilum (Hilum + PRHB)^14^.

### Clinical outcomes

All 900 patients were followed in our hospital. Clinical follow-up and management were independent of artificial intelligence assessment. The duration of follow-up was begun at the time of the initial tests and ended in October 2019. The primary composite endpoints in this study were admission or occurrence of HF with PH. PH was defined by elevated pulmonary artery pressure using RHC, or echocardiographic signs, including assessment of dilation of the RV, inferior vena cava size (diameter > 21 mm with decreased inspiratory collapse), elevated tricuspid regurgitation velocity (> 2.8 m/s)^15^, or clinical findings, including pretibial edema and abdominal fluid and jugular vein distension.

### Import data

Each case contains CXR images. We transformed all DICOM images into 512 × 512 resolution portable network graphic images with down sampling. All data were divided into 10 groups, 9 of the groups were used as a training and validation to create a model, and the rest were used to test the model so that the 900 total cases were split with 90 cases × 10 groups. Also, the images of the training dataset were augmented by using gamma correction, horizontal flipping, rotation and pixel shift. Then, we have done nested-cross validation (Supplemental Fig. S3) and tuned hyperparameters using grid-search.

### Deep learning model

**Figure 1:**
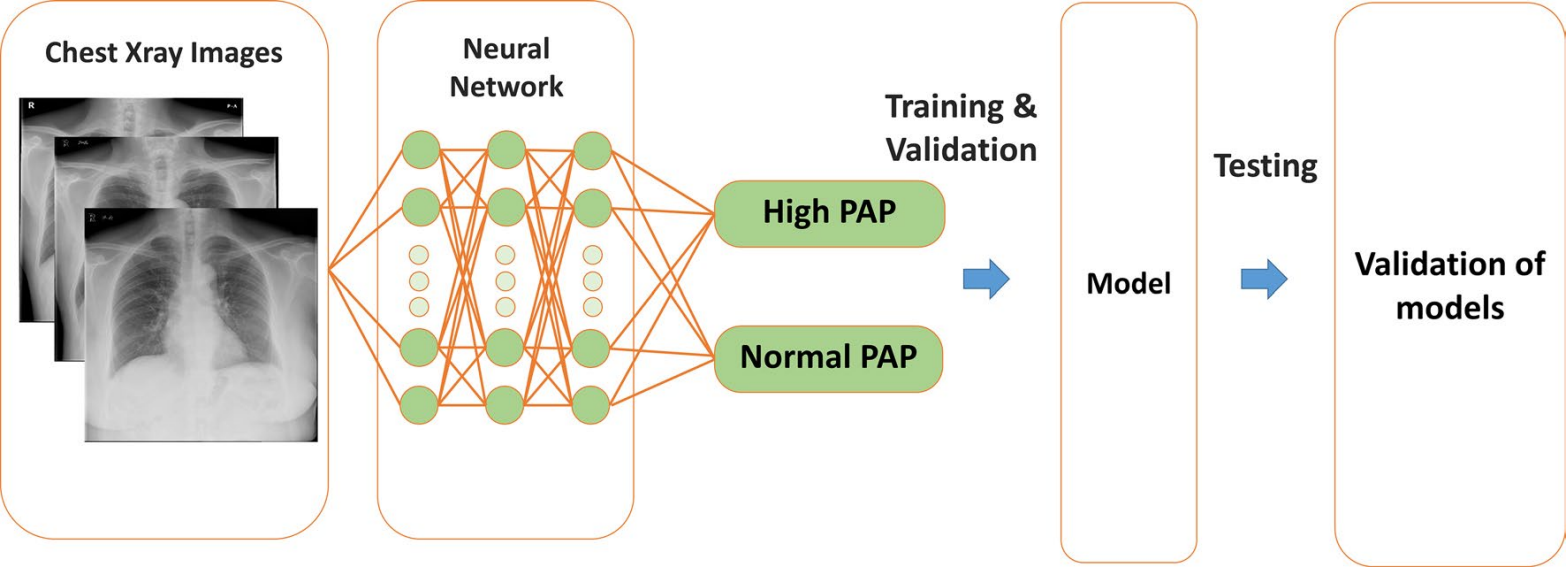
Neural network: An example of a convolutional network model for detection of pulmonary hypertension. PAP, pulmonary artery pressure.

The overall process is shown in Fig. 1. Ground truth data was consisted of two classes of high PAP (mean PAP > 20 mmHg) or low PAP (mean PAP ≤ 20 mmHg). We built a capsule-network-based model, with the addition of some residual blocks, to detect PH^16–18^. A capsule network is said to learn positional relations in images^19,20^. Residual blocks are helpful to prevent a vanishing gradient. Hence, it can train a deeper network. Each residual block contains two convolution layers, two batch normalizations, a rectified linear unit (ReLU), and a skip connection. Details are shown in Supplemental Fig. S4. The network consists of six residual blocks, six convolution layers, and six batch normalizations. All activation functions are set to ReLU functions. The highest elements in the likelihood vector are defined as the output label. The proposed network architecture is presented in Supplemental Fig. S5. We pre-trained the model using a CXR dataset, which is published by RSNA Pneumonia Detection Challenge in Kaggle (https://www.kaggle.com/c/rsna-pneumonia-detection-challenge). Then, we performed fine-tuning with the pre-trained model and nested tenfold cross-validation. The batch size was set to 16, and the Adam optimizer was used for training. We built the proposed network model on a computer (Xeon CPUs; Intel Corp. and Tesla P100 16 GB GPU; NVIDIA Corp.) with a Chainer (ver. 7.2.0) deep learning framework.

### Statistical analysis

Data are presented as mean ± SD. There was no patient excluded due to the lack of data in our analysis. The diagnostic performance of the DL algorithm and observers was evaluated using receiver operating characteristic (ROC) analysis and pairwise comparisons of the area under the ROC curve (AUC) according to the DeLong method^21^. Survival was estimated by means of the Kaplan–Meier method with the log-rank test. A median value of Hilmus (cutoff value: 104 mm) in CXR images was used to divide groups for the Kaplan–Meier analysis. Statistical analysis was performed using standard statistical software packages (SPSS software 21.0; SPSS Inc, Chicago, IL, USA, and MedCalc Software 17; Mariakerke, Belgium). Statistical significance was defined by p < 0.05.

## Results

### Clinical characteristics

The subject characteristics included in this study are shown in Table 1. The training cohort consisted of 900 patients with suspected PH. Blood pressure and heart rate at baseline were well-controlled in this cohort (heart rate: 74 ± 16 bpm and systolic blood pressure: 122 ± 22 mmHg). Subjects were divided into two groups with PH (PH group: 439 patients; mean age, 66 ± 14 years; 233 male) and without PH (non-PH group: 461 patients; mean age, 68 ± 12 years; 278 male). There were 215 patients with pre-capillary PH (mPAP > 20 mmHg and PAWP ≤ 15 mmHg) and 224 patients with post-capillary PH (mPAP > 20 mmHg and PAWP > 15 mmHg). The characteristics of the patients in the separate validation group is also summarized in the Table 1. There is no difference in the characteristics between the training cohort and the validation cohort.

**Table 1:** Baseline characteristics of the study population.

	Training Group	PH group	non-PH group	P value	Validation group
Number	900	439	461		90
Age, years	67 ± 13	66 ± 14	68 ± 12	0.004	64 ± 13
Male, n (%)	511(57)	233(53)	278(60)	0.014	54(59)
HR, bpm	74 ± 16	76 ± 16	73 ± 15	0.001	73 ± 15
Height, m	1.59 ± 1.0	1.58 ± 0.9	1.59 ± 1.0	0.137	1.59 ± 1.0
Weight, kg	60 ± 15	61 ± 17	59 ± 13	0.010	60 ± 14
SBP, mmHg	122 ± 22	122 ± 22	122 ± 21	0.364	121 ± 20
DBP, mmHg	69 ± 14	69 ± 15	70 ± 14	0.321	71 ± 15
CI, L/min/m^2^	2.8 ± 0.8	2.8 ± 0.9	2.7 ± 0.7	< 0.001	2.8 ± 0.9
mean PAWP, mmHg	12.4 ± 6.4	16.5 ± 6.2	8.4 ± 3.4	< 0.001	12.8 ± 6.4
mean PAP, mmHg	21.3 ± 8.9	28.2 ± 7.8	14.8 ± 2.9	< 0.001	21.6 ± 8.4
mean RAP, mmHg	5.9 ± 4.1	7.8 ± 4.3	4.0 ± 2.9	< 0.001	6.1 ± 3.8
PVR, Wood unit	2.2 ± 2.0	2.8 ± 2.6	1.6 ± 0.8	< 0.001	2.2 ± 1.8

Data are presented as number of patients (percentage), mean ± SD.

*HR* heart rate; *BP* blood pressure; *BMI* body mass index; *AF* atrial fibrillation; *CO* cardiac output; *CI* cardiac index; *PAWP* pulmonary arterial wedge pressure; *PAP* pulmonary artery pressure; *RAP* right atrial pressure.

### Image selection

We examined various image selections in our analysis. We prepared three datasets; Dataset 1: no exclusion criteria (n = 900). Dataset 2: excluded for low image quality from Dataset 1 (n = 833). Dataset 3: excluded for wire, pacemaker, and any artificial materials from Dataset 2 (n = 748). We compared the diagnostic ability of AI from learning in 3 different datasets. In the results, there were no significant differences among AI algorithms from 3 datasets on nested tenfold cross validation (Supplemental Fig. S6. Dataset 1: AUC: 0.69 ± 0.05; Dataset 2: AUC: 0.72 ± 0.07; and Dataset 3: AUC: 0.66 ± 0.05, all comparisons were p > 0.05). Thus, we determined Dataset 1 as a final analysis cohort.

### Detection for pulmonary hypertension

**Figure 2:**
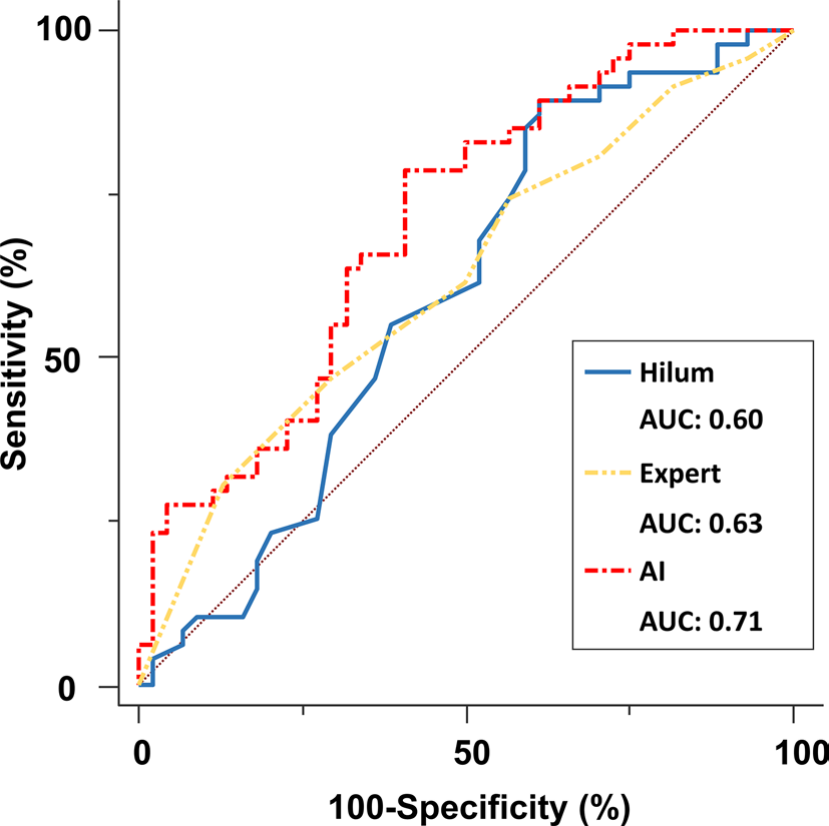
Diagnostic ability for pulmonary hypertension: The area under the curve by AI algorithms for detection of pulmonary hypertension was significantly higher than the AUC by measurement of CXR images and human observers.

Results of the ROC analysis used to assess the diagnostic ability for detecting the PH in the separate validation cohort are shown in Fig. 2. The measurements of CXR images with largest AUC was Hilum (Hilum: AUC = 0.60, PRHB: AUC = 0.58, Hilum/chest ratio: AUC = 0.60, Hilum + PRHB: AUC = 0.59). We compared the AUCs using human observers, measurements of CXR images, and artificial intelligence for detection of PH (normal mean PAP and high mean PAP). The highest diagnostic ability was AI (AUC: 0.71). For detection of PH, the AUC by the AI algorithm was significantly higher than the AUC by measurement of CXR images and human observers (0.71 vs. 0.60 and vs. 0.63, all comparisons were p < 0.05). Importantly, the negative predictive value (NPV) of AI algorithm for detecting PH was 95.0% using the cut off value of 0.42 for AI estimated probability (Supplemental Table S1). To check the generalizability of our results, we gathered an external validation group of 55 patients (Supplemental Table S2), the AUC by the DL algorithm was 0.70. The AUC in this group was similar to that of the original cohort.

### Prognosis in patients with AI predicted PH vs. without AI predicted PH

**Figure 3:**
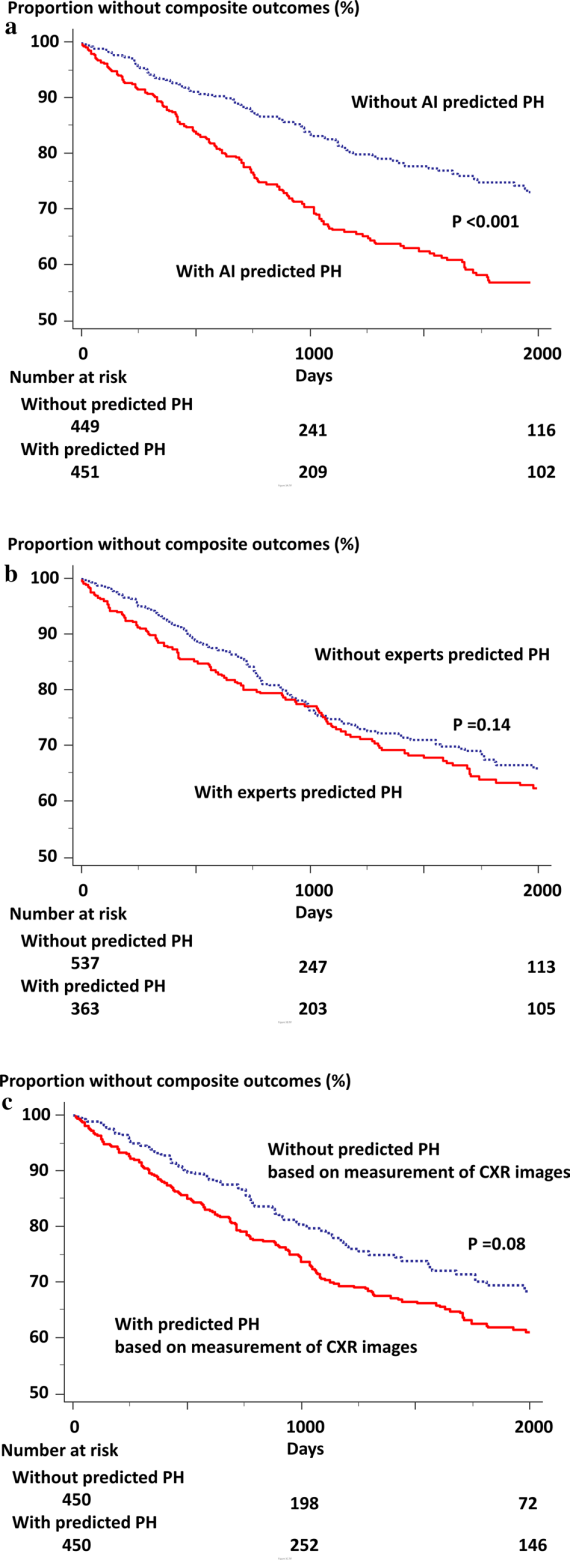
Incidence of composite outcomes in patients stratified by AI classification (**a**), human observers (**b**), and measurement of CXR images (**c**). The composite outcomes were admission or occurrence of HF with PH. Of these patients with AI predicted PH, 37% patients had the composite outcomes (red line) during follow-up. Of these patients without AI predicted PH, 22% patients had the composite outcomes (blue line) during follow-up.

The subject characteristics of patients with AI predicted PH and without AI predicted PH are shown in Supplemental Table S3. In patients with predicted PH, 167 patients (37%) had the composite outcomes over a median follow-up period of 5.9 years (Fig. 3a, red line). Of these patients without predicted PH, 97 patients (22%) had the composite outcomes over a median follow-up period of 7.7 years (Fig. 3a, blue line). This represented a twofold risk of future events when the AI algorithm defines the CXR as abnormal (hazard ratio: 1.9, 95% confidence interval: 1.5–2.4, p < 0.001). Interestingly, there was no significant difference in Kaplan-Meir plots between two groups defined by human observers (Fig. 3b, p = 0.14) and measurement of CXR images (Fig. 3c, p = 0.08).

### Assessment of heat map

**Figure 4:**
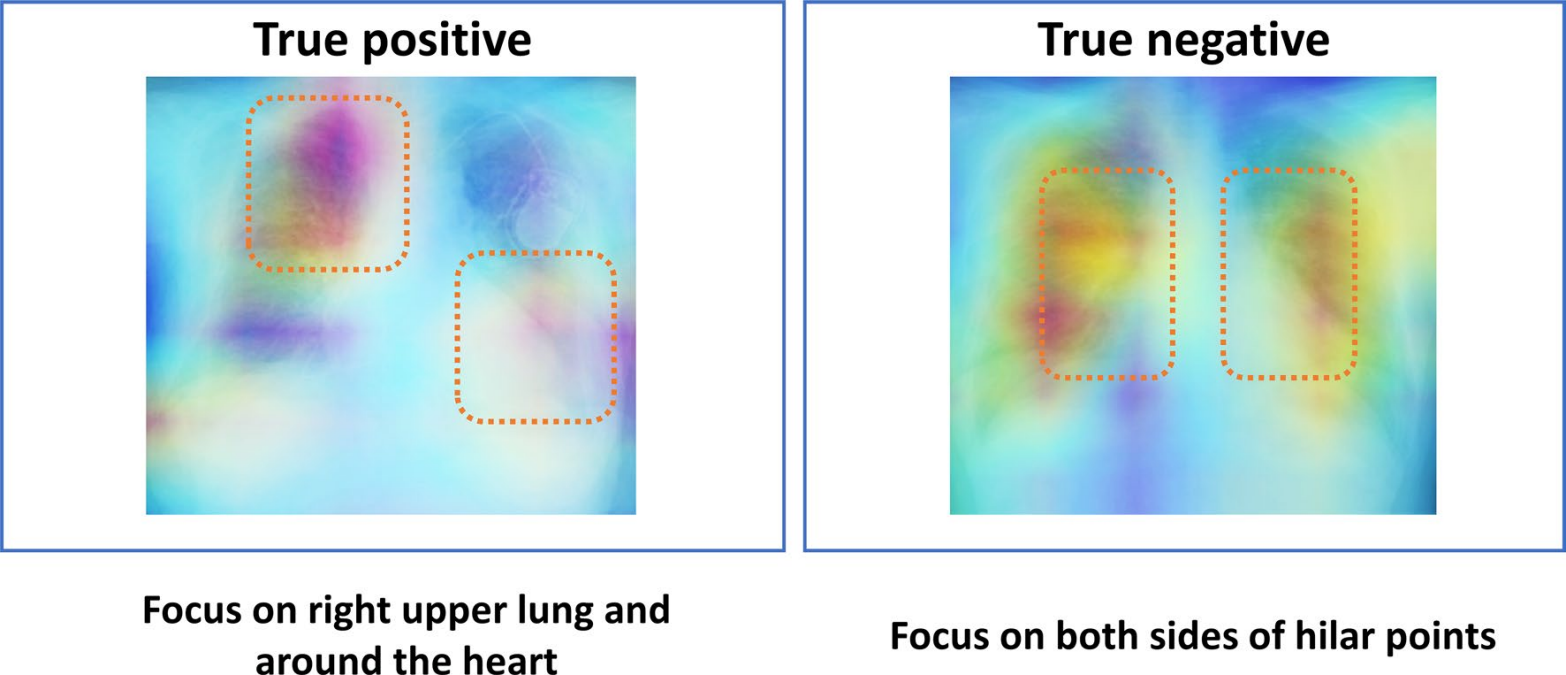
Examples of grad-CAM visualizations. In the elevated pulmonary artery pressure (PAP) group, the focus by AI tended to be on the right upper lung area and the area around the heart. In the normal PAP group, the focus by AI tended to be on the both sides of hilar points.

To help understand the AI assessment, we have analyzed the images where AI was focused using Grad-CAM^22^. Grad-CAM revealed that our model focused on the specific areas of CXR. To clarify the areas, we superimposed heat map images in 10 true positive and true negative cases in Fig. 4. In the elevated PAP group, the focus by AI tended to be on the right upper lung area and the area around the heart. In the normal PAP group, the focus by AI tended to be on the both sides of hilar points. In general, the right upper pulmonary field is a common site of focal congestion (e.g. mitral regurgitation), enlargement of the heart can suggest increased pulmonary artery pressure, and both hilar areas are also important in cases with elevated pulmonary artery pressure. Thus, we believed the resulting AI model was able to appropriately discern differences in CXR images and the findings were consistent with our previous knowledge.

## Discussion

We demonstrated that DL algorithm is an objective method, and its discrimination was similar to that of assessment by experts and manual measurements. In addition, because of the high negative predictive value using a particular threshold, undergoing unnecessary RHC might be reduced using the AI algorithm. Importantly, we note that in patients with a network-predicted PH, there was 2-times increase in risk of future heart failure hospitalization with PH compared with patients without a network-predicted PH. On the other hand, there was no significant difference in Kaplan-Meir plots between two groups defined by human observers and measurement of CXR images. This result suggests that the AI algorithm can obtain a new insight using a standard economical method. To our knowledge, this is the first study to demonstrate that the AI algorithm adds new information to CXR for the prediction of PH confirmed by invasive methods.

### Comparison with previous analysis

Previously, CXR is a simple and economical method that is available globally. Thus, this technique is considered a useful tool to check patients with suspected elevated PAP^23^. A recent study showed measurements of CXR can lead to identifying more subjects suffering from undiagnosed PH^14^. However, the AUC was limited as 0.60–0.62. Moreover, there were several methods including laboratory data, electrocardiogram, and physical examinations for detection of PH^24–27^. In previous studies, the number of invasive data was limited, and the accuracy was also limited as AUCs up to 0.65 for these methods. Our results of CXR measurements are consistent with those works. In the view point of reproducibility, automated assessment is needed to obtain quantitative results without any user interaction including measurements. Our results demonstrate that an AI model can be trained to estimate PH on CXR images. We believe this study is a pilot study leaning towards the possibility of applying a DL algorithm in the clinical assessment of pulmonary hypertension.

### Additional knowledge from artificial intelligence for Chest X-ray

The specific CXR characteristics used by the convolutional neural network to classify individuals as having PH are not well known because of a “black box” algorithm. We suspect it is detecting the known pathological effects of PH on the CXR. According to the results of heat map analysis, AI assessment mainly focused on the heart and lung areas. The AI algorithm might be tracing the process of human knowledge. The use of AI may be extended beyond the capacity of human knowledge in the future.

### Clinical implications

Assessment of PH using the AI algorithm is an objective method, and its discrimination was similar to that of assessment by experts and manual measurements. CXR can be used as an inexpensive, standardized, universal test. If many patients can access an inexpensive, reasonable test for PH, individual patients could benefit from early effective therapies. For examples, connective tissue disease is a high-risk group of developing pre-capillary PH and one of the most suspected histories of PH^1,13^. This method may allow us to identify more patients with possible PH in regions lacking sufficient imaging facilities. In the clinical setting, it is not so difficult to detect post-capillary PH caused by left heart failure. On the other hand, early detection of pre-capillary PH is also important, because it is often misdiagnosed, and treatment of pre-capillary PH may differ from post-capillary PH. Future studies involving larger numbers are required to assess the AI models for the differentiation of pre- and post-capillary PH.

In this study, AI tool was tested in patients who have already had an echocardiographic study and CXR indicating suspected PH. Because it is impossible to use the RHC for the patients without suspected cardiovascular diseases due to the ethical issues. Thus, we were unable to claim any value of the AI assessment in screening patients for PH based on clinical signs alone. AI assessment may be considered an option to check the necessity of RHC in patients with suspected PH, with a negative predictive value of 95%. However, further validation is necessary to determine the feasibility of CXR utilized in a stand-alone manner.

### Limitations

First, the number of patients was modest. Although DL algorithms require thousands of patients, our “prognosis” data strongly support the new insight for this approach. Second, we did not make the model to predict specific types of PH (e.g. normal, pre-capillary PH, post-capillary PH), because the number of patients is too small to make a feasible AI model after classification. They should be evaluated further by echocardiography and cardiac catheterization in the referral centers. Third, this population did have an indication for RHC, it is a high-risk population. The AI algorithm should be tested in a more general screening setting for PH. Forth, the accuracy of AI algorithm was modest. Fifth, due to the limited diagnostic performance, we thought this approach may be more suited to detect elevated pulmonary artery pressure as the first-line test especially in a situation where echocardiography accessibility is limited. However, further clinical trials are needed to assess the clinical feasibility for this method in a real-world setting. Regarding these limitations, this study is a preliminary work and we believe that this report can serve as an impetus for a future large multi-center study.

## Conclusions

Applying AI to the CXR (a conventional, universal, low-cost test) is a potential tool to detect PH. In addition, this tool may also have a potential to identify individuals at increased risk for its future events. However, this preliminary work suggests that applying AI to the CXR in high risk groups has limited performance in identifying elevated PAP. It should be considered that it is premature to include this technology in the current guidelines.

## Supplementary information

Supplementary Information 1.

Supplementary Information 2.

## Data Availability

Data are available upon reasonable request.

## Code availability

The code has been uploaded in GitHub (10.5281/zenodo.4067277).
