# Supplementary material for: Deep learning to predict elevated pulmonary artery pressure in patients with suspected pulmonary hypertension using standard chest X ray
Source: Sci Rep. 2020 Nov 17;10:19311. doi: 10.1038/s41598-020-76359-w (PMC7672097; doi:10.1038/s41598-020-76359-w)
Supplement: Supplementary file 1 — Supplementary Information 1. [file 41598_2020_76359_MOESM1_ESM.pdf]

Deep Learning to Predict Elevated Pulmonary Artery Pressure in Patients with Suspected Pulmonary Hypertension Using Standard Chest X Ray

Brief title: Deep Learning for Pulmonary Hypertension

Kenya Kusunose, MD, PhD[1], Yukina Hirata, PhD[2], Takumasa Tsuji BSc[3], Jun'ichi Kotoku, PhD[3], Masataka Sata, MD, PhD[1]

[1]Department of Cardiovascular Medicine, Tokushima University Hospital, Tokushima, Japan

[2]Ultrasound Examination Center, Tokushima University Hospital, Tokushima, Japan

[3]Department of Radiological Technology, Graduate School of Medical Care and Technology, Teikyo University, Tokyo, Japan

Supplemental Figure legends

Supplemental Figure 1: Flow chart of patient recruitment

Supplemental Figure 2: We measured the widening of hilum from the most lateral visible border of hilum to the other lateral border (Hilum) and projection of the right heart border (PRHB) that consists of the distance from the right visible border of the right side of the heart up to the midline of the thorax.

Supplemental Figure 3: Import Data: A total of 900 cases were split into 90 cases × 10 groups. Nested 10-fold cross-validation was employed to show a model performance that we used to detect pulmonary hypertension.

Supplemental Figure 4: Architecture of the residual block: The network consists of six residual blocks, six convolution layers, and six batch normalizations. All activation functions are set to ReLU functions.

Supplemental Figure 5: Architecture of the Capsule Residual Network: We performed fine-tuning with the pre-trained model and nested 10-fold cross-validation.

Supplemental Figure 6: Averaged AUC between models on nested 10-fold cross validation.

Supplemental Figure 1.

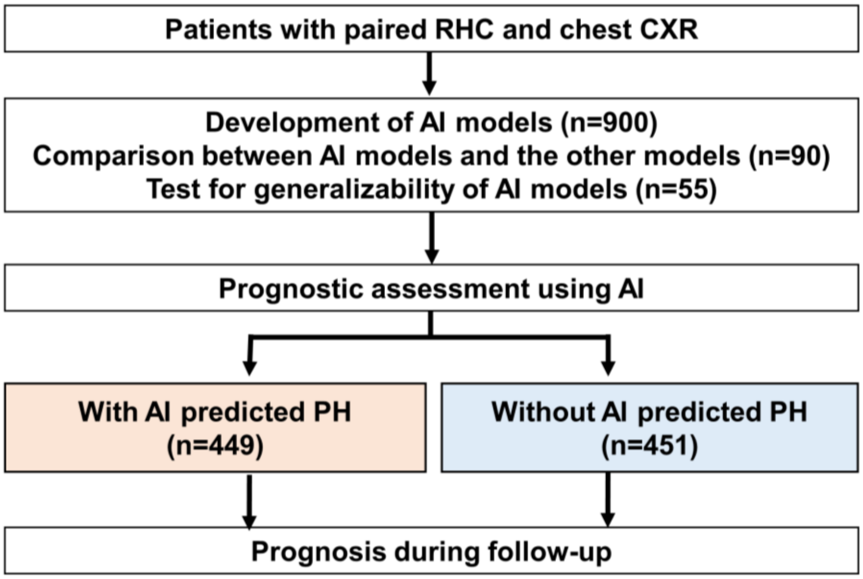

Supplemental Figure 2.

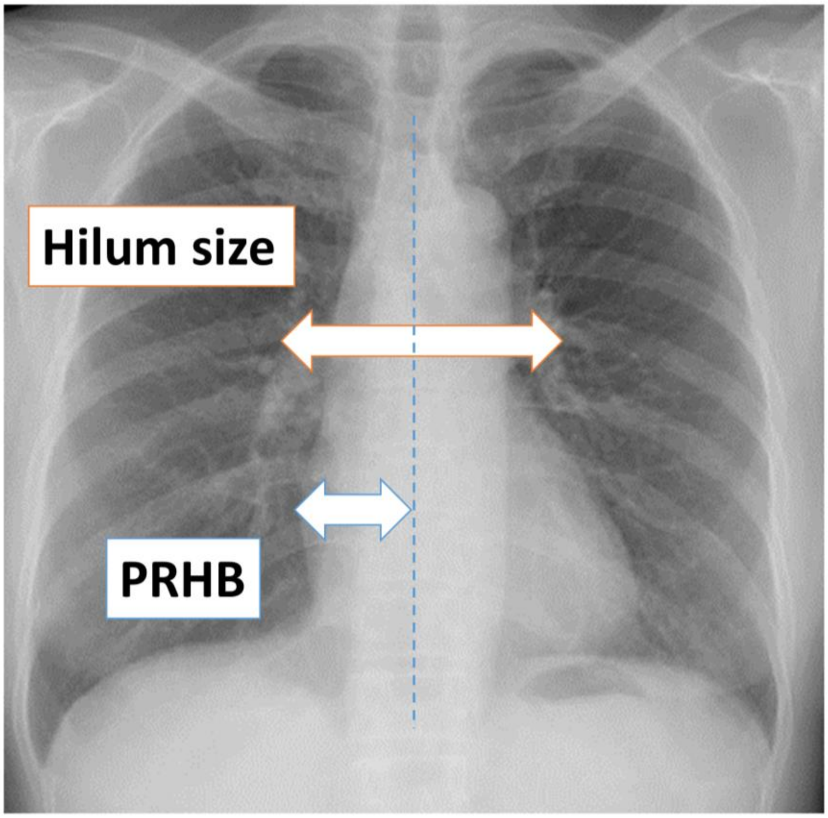

Supplemental Figure 3.

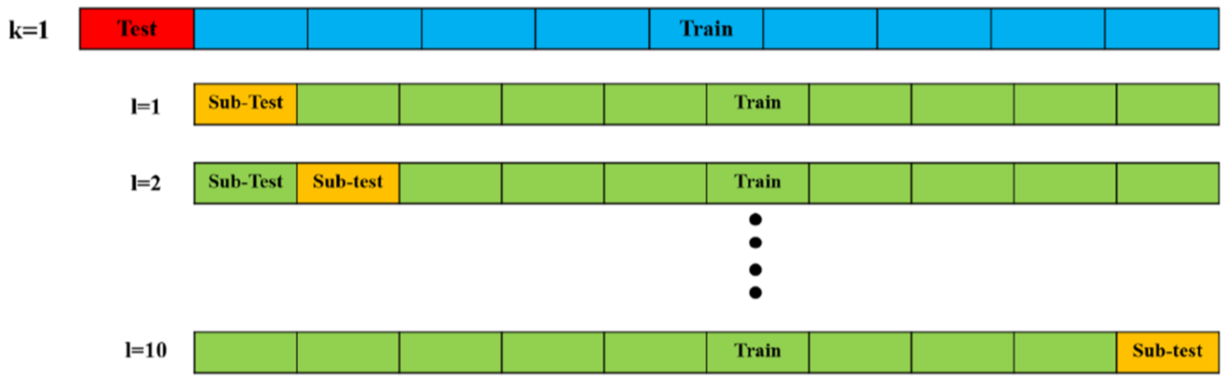

Supplemental Figure 4.

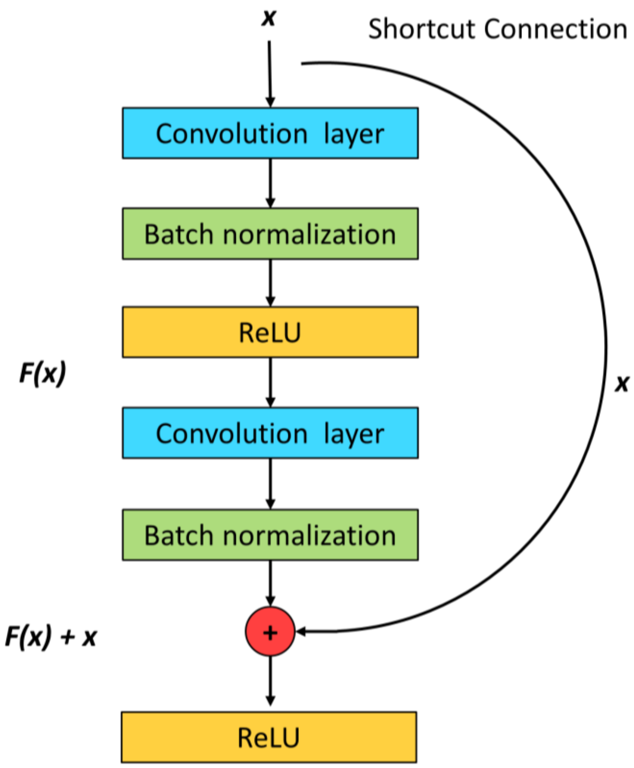

Supplemental Figure 5.

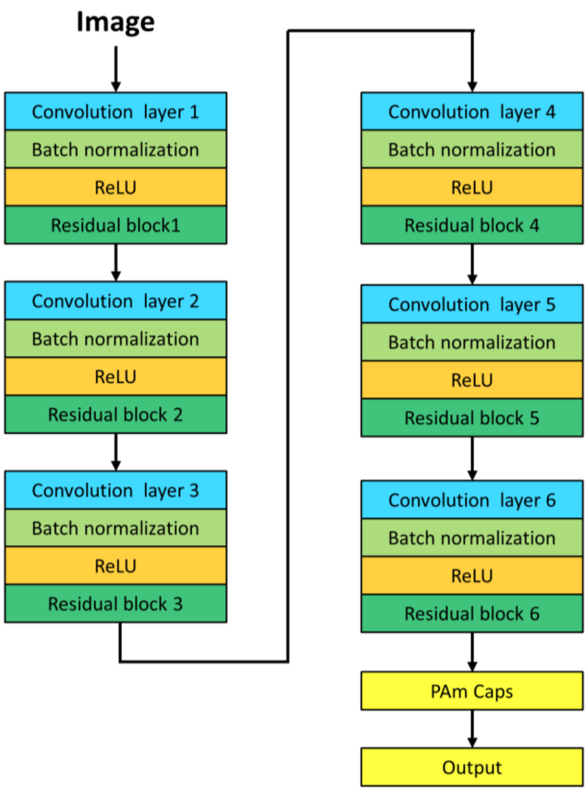

Supplemental Figure 6.

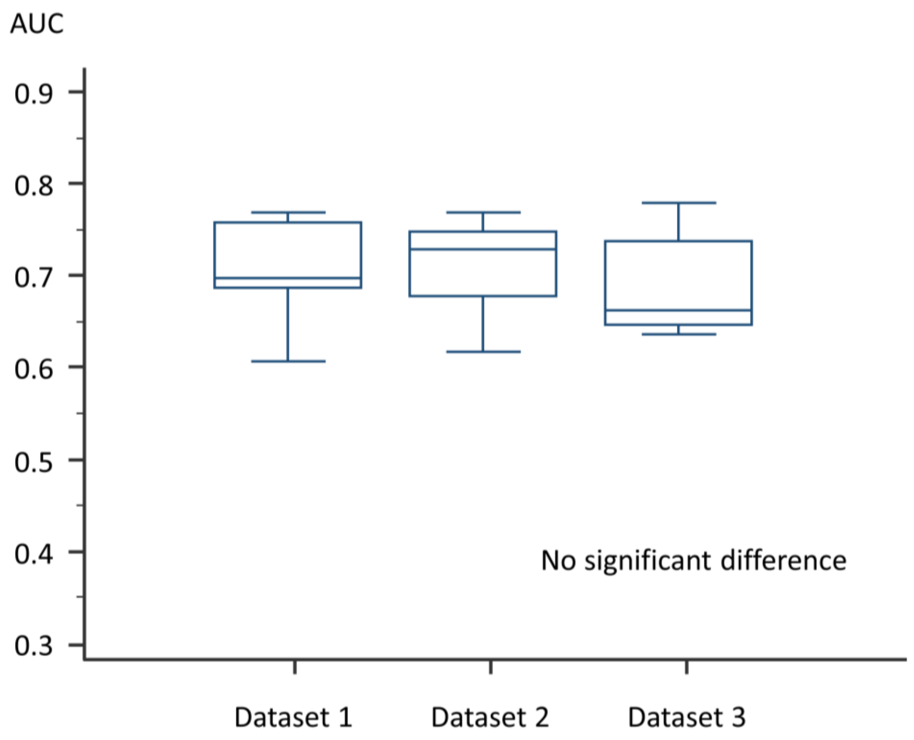

Supplemental Table 1: Cross tabulation for detecting PH

	Actual PH	Actual no-PH	ALL
Predicted PH	43	27	70
Predicted no-PH	1	19	20
ALL	44	46	90

Sensitivity: 98%

Specificity: 41%

Positive predictive value: 61%

Negative predictive value: 95%

Supplemental Table 2: Independent validation cohort.

	Independent validation cohort
Number	55
Age, yrs	67±14
Male, n (%)	32 (58)
HR, bpm	70±12
SBP, mmHg	118±28
CI, L/min/m^2	2.9±0.6
mean PAWP, mmHg	14.3±6.5
mean PAP, mmHg	25.0±11.1
High mean PAP (>20 mmHg)	34 (62)
PVR, Wood unit	2.5±2.1

Data are presented as number of patients (percentage), mean ± SD. Abbreviations: See Table 1.

Supplemental Table 3: Baseline characteristics between with and without AI predicted PH.

	With AI predicted PH	Without AI predicted PH	P value
Number	451	449	
Age, yrs	67±14	67±13	0.72
Male, n (%)	226 (50)	285 (63)	<0.001
HR, bpm	74±16	70±14	<0.001
Body surface area, m	1.6±0.2	1.6±0.2	0.46
SBP, mmHg	120±22	123±21	0.05
DBP, mmHg	69±15	70±14	0.29
CO, L/min/	4.4±1.5	4.5±1.4	0.12
mean PAWP, mmHg	13.5±6.8	11.2±5.8	<0.001
mean PAP, mmHg	24.0±9.8	18.6±6.9	<0.001
mean RAP, mmHg	6.7±4.4	5.0±3.5	<0.001
PVR, Wood unit	2.6±1.3	1.7±1.3	<0.001

Data are presented as number of patients (percentage), mean ± SD. Abbreviations: See Table 1.
